# Previously Unrecognized Land‐Use Control on Deep Soil CO_2_ Sink

**DOI:** 10.1111/gcb.71119

**Published:** 2026-09-28

**Authors:** Kenneth Tetteh, Mostafa Abdollahpour, Leopold Sauheitl, Georg Guggenberger, Kazem Zamanian

**Affiliations:** ^1^ Institute of Earth System Sciences, Section Soil Science Leibniz University Hannover Hannover Germany

**Keywords:** climate change, CO_2_ production, CO_2_ transport, hydrological modeling, land use, process modeling (DeepCO_2_), soil CO_2_ storage

## Abstract

Land‐surface models commonly assume that soil CO_2_ production is rapidly transferred out of the soil profile, effectively equating respiration with near‐surface signals. However, persistent subsoil CO_2_ enrichment despite limited in situ production reflects bidirectional exchange of topsoil‐respired CO_2_, suggesting that the surface‐centric assumption may not be universally valid. Here we combine year‐round, multi‐depth field observations with process‐based modeling (DeepCO_2_) to quantify the effects of land use (croplands, forests, and forest‐cropland edges) and climate on soil CO_2_ production‐transport dynamics. We reveal pronounced vertical decoupling of soil CO_2_, with deep CO_2_ (> 1 m) concentrations up to two orders of magnitude above surface concentrations and 9–17 times greater than can be explained by in situ production alone, revealing profile‐scale production‐transport imbalance. Forest soils promote ~2.3‐fold greater deep CO_2_ accumulation than cropland and edge systems, driven by enhanced macroporosity and stronger advective coupling. Seasonal climate forcing regulates these regimes, with winter cold spells suppressing exchange, whereas spring–summer warming amplifies CO_2_ source strength originating from otherwise “protected” subsoil organic carbon, particularly in cropland and edge soils. These findings demonstrate that a substantial fraction of topsoil‐respired CO_2_ is redistributed below the soil‐atmosphere boundary and retained within the subsurface, forming a land‐use‐sensitive CO_2_‐C sink. Surface‐based CO_2_ assessment therefore reflects a transport‐limited, boundary‐conditioned signal rather than a direct proxy for profile‐scale production, and alone cannot resolve the full partitioning of soil‐respired carbon. Incorporating depth‐dependent production‐transport coupling is therefore essential for accurately representing terrestrial carbon allocation and land‐climate feedbacks under ongoing land‐use change and climate warming.

## Graphical abstract

Transport‐limited, boundary‐conditioned bidirectional exchange of topsoil‐respired CO_2_ drives CO_2_ accumulation in less bioactive subsoils in concentrations that cannot be explained by in situ production alone and far exceed atmospheric boundary concentrations, revealing a production‐transport imbalance and a potential land‐use‐sensitive pathway for deep‐soil CO_2_ retention.

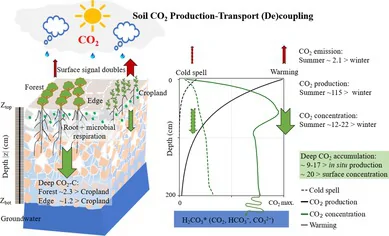

## 1 Introduction

Deep (> 1 m) soil carbon processes are increasingly recognized as critical but underrepresented components of the global carbon budget, governing soil‐atmosphere CO_2_ exchange and the persistence of terrestrial carbon under climate change (IPCC 2023; Dong et al. 2026). While soil carbon pools comprise both organic carbon (SOC) and inorganic carbon (SIC), their climatic relevance is mediated primarily through CO_2_, which acts as a central mobile intermediate linking biological production, physical redistribution, and geochemical stabilization across the soil profile (Schlesinger and Andrews 2000; Zamanian et al. 2016). CO_2_ produced by microbial decomposition of SOC and root respiration accumulates in soil pore spaces, emits to the atmosphere, migrates downward, or dissolves into soil water, where it regulates acid–base equilibria, mineral weathering, and carbonate formation (Kuzyakov 2010; Hardie et al. 2009; Davidson and Trumbore 1995). These coupled processes form a vertically integrated CO_2_ transformation continuum that is highly sensitive to soil temperature, moisture, aeration, and diffusivity, collectively determining whether respired carbon is rapidly emitted or retained at depth as a long‐term sink (Hao et al. 2022; Min et al. 2020; Zamanian et al. 2016; Richter and Billings 2015).

Despite this central role, mechanistic understanding of deep (> 1 m) soil CO_2_ dynamics remains limited, as more than 80% of observational and modeling studies are confined to the upper 30–50 cm, overlooking subsurface production, transport, and seasonal redistribution critical for identifying source versus efflux‐sink partitioning dynamics (Stewart et al. 2024; Tian et al. 2010). Land use and climate further influence these dynamics by regulating rooting depth, organic matter inputs, soil structure, and gas‐water transport properties, thereby exerting strong controls on topsoil respired CO_2_ source strength and its exchange between atmosphere and subsoil (Dong et al. 2026; Giardina et al. 2014; Heinze et al. 2018; Leiber‐Sauheitl et al. 2014). However, surface‐based assessments alone fail to resolve vertical CO_2_ gradients under non‐steady‐state conditions, limiting mechanistic understanding of subsoil CO_2_ enrichment and robust evaluation of land‐use effects on CO_2_‐C source‐sink dynamics (Stewart et al. 2024; Elberling 2003; Sánchez‐Cañete et al. 2013). More fundamentally, interpretations derived from single spatial or temporal scales are inherently confounded, necessitating model‐data integration frameworks that reconcile production, transport, and storage processes across scales (Reichstein and Beer 2008).

Here, we combine depth‐resolved field observations with HYDRUS‐based mechanistic modeling to quantify how atmospheric forcing, soil moisture, and temperature regulate CO_2_ production and transport across contrasting land uses (Šimůnek and Suarez 1993; Suarez and Šimůnek 1993; De Pue et al. 2019). We focus on CO_2_ production and redistribution as coupled processes governing source strength, atmospheric exchange, and subsoil accumulation. This framework resolves the bidirectional fate of soil‐respired carbon and provides a process‐based foundation for improved representation of terrestrial carbon allocation and land‐climate feedbacks under ongoing land‐use change and warming.

We tested three hypotheses across forests, croplands, and their transitional forest‐cropland boundaries (edges): (i) Integrating field observations with mechanistic modeling of biological, thermal, and hydrological processes is expected to resolve the fate of soil‐respired CO_2_ more accurately than surface‐based approaches by capturing the dynamic, bidirectional pathways and controls governing CO_2_ exchange across climatic variables and surface‐subsurface interactions. (ii) Land‐use‐specific differences in belowground substrate availability, rooting patterns, temperature, and water‐air partitioning are expected to influence the depth distribution of CO_2_ production, with relatively denser and deeper‐rooted forest soils expected to support greater deep inputs of respired carbon. (iii) Daily to seasonal climatic forcing (rainfall, evapotranspiration, and temperature) is expected to influence land‐use contrasts in production‐transport (de)coupling, where seasonal shifts in soil temperature and moisture, together with land‐use‐specific vegetation and soil structure, regulate macroporosity‐driven surface‐subsurface connectivity, control effective diffusivity (D_e_), and determine the magnitude and fate of soil‐respired carbon.

## 2 Methods

### 2.1 Study Area and Climatic Conditions

The study was conducted in the Fuhrberger Feld catchment (52.52° N, 10.05° E), northern Germany's largest groundwater protection area, located ~30 km northeast of Hannover (Figure S1a). The region has a temperate humid climate with a mean annual temperature of 8.9°C and precipitation of ~680 mm. Soils are predominantly sandy (> 90% sand; Table S1), classified as Podzols and Gleysols, overlying an unconfined aquifer with a fluctuating water table (2–3 m depth), providing a suitable setting for quantifying deep carbon dynamics under shared hydrogeological context.

The experimental layout comprised three landscape clusters, each containing cropland (A1–A3), adjacent forest (F1–F3), and transitional edge sites (E1–E3) (Figure S1b). This triadic design enables direct comparison of CO_2_ production and transport processes across contrasting land uses and their interfaces within a shared hydrogeological context, reducing climatic and edaphic variation among sites and allowing evaluation of land‐use effects on vertical CO_2_ dynamics.

### 2.2 Field Instrumentation and Site Setup

To assess CO_2_ production and transport across land uses, we established vertically stratified monitoring at six depths: 0–10, 10–30, 30–50, 50–100, 100–150, and 150–200 cm (Figure 1). These intervals represent (i) biologically active topsoil (0–10 and 10–30 cm), (ii) the upper subsoil (30–50 cm), (iii) root‐diminished mid‐vadose (50–100 and 100–150 cm), and (iv) the capillary fringe (150–200 cm), capturing contrasting zones of biogeochemical activity and gas‐water transport.

**FIGURE 1:**
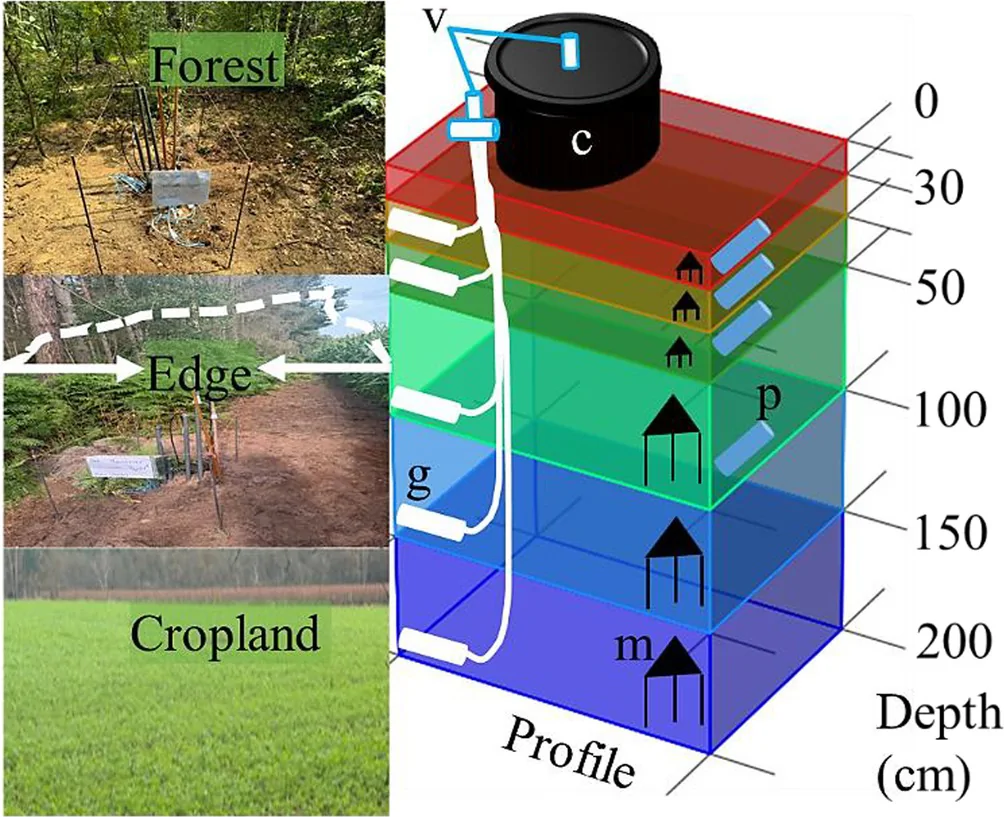
Schematic of instrumentation across land‐use profiles (0–200 cm). (c) CO_2_ chambers for efflux measurement (0–10 cm); (g) EcoTech gas samplers for deep CO_2_ sampling (30–200 cm); (m) TRIME‐PICO TDR sensors and/or SMT‐100 probes for soil water content and temperature; (p) precision tensiometers for soil water tension. Each CO_2_ sensor and chamber is equipped with three‐way valves (v) for controlled syringing.

Soil water content, temperature, and electrical conductivity were monitored with TRIME‐PICO TDR (IMKO GmbH, Ettlingen, Germany) or alternatively SMT‐100 probes (UGT GmbH, Müncheberg, Germany), installed at 10, 30, 50, and 100 cm with soil slurry to ensure adequate contact or via augered boreholes at 150 and 200 cm to minimize preferential flow. Tensiometers (GMH 3100, GHM Messtechnik GmbH; formerly Greisinger, Regenstauf, Germany) tracked soil water tension (0–10, 10–30, 30–50, 50–100 cm depth). The 0–10 and 10–30 cm measurements resolved surface energy and water exchanges.

CO_2_ concentrations were monitored using EcoTech glass gas samplers (EcoTech Umwelt‐Messsysteme GmbH, Bonn, Germany; water entry pressure~49 kPa) at four depths (10–200 cm) and static chambers (10 cm air space, 5.5 cm radius) at the surface to capture efflux. Chambers remained open between measurements to prevent pressure artifacts. Across nine plots (three each in arable, forest, and edge zones), 45 subsurface CO_2_ sampling points, 9 surface CO_2_ chambers, 54 temperature and moisture sensors, and 36 tension sensors (Figure 1) were installed by April 2024. Baseline soil physical, biological, and chemical properties were determined from intact and disturbed cores at four replicates per depth defined above.

### 2.3 Sampling Strategy and Temporal Monitoring

To minimize post‐installation artifacts (altered diffusivity, root dieback, solute pulses), soil gas, water and temperature monitoring began in August 2024, 3 months after sensor deployment, allowing structural and biogeochemical equilibration. Soil gas was sampled monthly from each depth using EcoTech samplers. Valves were sealed for 15 min to permit in situ CO_2_ accumulation, after which 15 mL samples were extracted into pre‐evacuated Exetainers; a second draw 20 min later resolved short‐term production rates. Soil CO_2_ efflux was measured concurrently using static chambers under ambient conditions, linking subsurface production with soil‐atmosphere interface emissions for model calibration. Simultaneous surface (0–10 cm) and subsurface temperature measurements enabled thermal correction.

This design integrating depth‐resolved gas, moisture, and thermal monitoring with seasonal biological metrics captured a near annual cycle (Aug 2024–Jun 2025) of coupled CO_2_ production and transport and accumulation dynamics across contrasting land uses.

### 2.4 Microbial and Root Biomass and Soil Physico‐Chemical Properties Measurements

To parameterize CO_2_ production and transport models, depth‐resolved measurements of soil microbial biomass and root distribution were conducted across five seasonal campaigns, aligned with major phenological phases, while physical and hydraulic properties (bulk density, texture, hydraulic conductivity) were sampled once during the installation campaign.

At each campaign, soil cores were taken at 0–30, 30–50, 50–100, and 100–200 cm. Roots were separated by wet sieving (0.5 mm), rinsed, oven‐dried at 60°C, and weighed to determine dry biomass (Freschet et al. 2021). Subsamples were analyzed for microbial biomass carbon (MBC) via chloroform fumigation‐extraction (Brooks et al. 1997), with quantification of extracted carbon using a TOC analyzer (Elementar Analysensysteme GmbH, Langenselbold, Germany), and for soil organic carbon (SOC) using an elemental analyzer (Elementar Analysensysteme GmbH, Langenselbold, Germany). Soil pH was measured potentiometrically in a 1:2.5 (m:v) soil‐solution suspension (Figure S2), and texture was determined by pipette and sieving methods (Gee and Or 2002), classifying particles as clay (< 0.002 mm), silt (0.002–0.05 mm), and fine sand (0.05–2.0 mm) (Table S1).

### 2.5 Hydraulic Properties Measurements

Soil water retention and unsaturated hydraulic conductivity were determined on intact 250 cm^3^ cores using the HYPROP evaporation method (METER Group GmbH, Munich, Germany). During controlled evaporation (~20°C), matric heads (1.25, 3.75 cm) and mass loss were continuously monitored, with volumetric water content calculated gravimetrically and hydraulic parameters derived from coupled mass‐pressure evolution. The dry range (pF > 3.5) was characterized by dewpoint potentiometry (WP4C, METER Group) on < 2 mm air‐dried fractions. HYPROP‐WP4C datasets were merged to generate full soil water retention curves using LABROS SoilView Analysis software (version 5.3.0.0; HYPROP, METER Group GmbH, Munich, Germany).

Saturated hydraulic conductivity (*K*_*s*_) was measured on 100 cm^3^ undisturbed cores via the falling‐head method (3 replicates × 5 runs per depth). Bulk density (*ρb*) was derived from oven‐dry mass (105°C) and core volume, and total porosity (ϕ) estimated as follows:

ϕ=1−ρb/ρs

where *ρb* is bulk density and *ρs* particle density (g cm^−3^).

This integrated dataset provided vertical and seasonal resolution of biological and physicochemical parameters critical for modeling soil CO_2_ dynamics under contrasting land uses.

### 2.6 DeepCO_2_ Modeling Framework for C‐CO_2_ Transformation and Transport

To resolve how soil organic carbon (SOC) mineralization translates into CO_2_ production and vertical transport, this study introduces the DeepCO_2_ modeling framework within HYDRUS‐1D (Šimůnek et al. 2006, 2008). The framework couples variably saturated water flow, heat transport, root and microbial respiration, and gas‐phase CO_2_ diffusion–advection, enabling depth‐resolved simulation of carbon turnover under transient hydrothermal forcing. Unlike surface‐centric approaches that capture only near‐surface signals, DeepCO_2_ resolves subsurface CO_2_ generation, redistribution, and retention, linking biological kinetics to physical transport processes.

DeepCO_2_ integrates three dynamically coupled submodules; (i) rainfall‐driven water flow (“make‐it‐rain”), temperature‐driven heat transfer (“warm‐it‐up/cool‐it‐down”), and biophysically regulated CO_2_ production‐transport (“CO_2_ pro‐trans”), with all three submodules driven by daily meteorological forcing. This coupling captures (i) rapid matric potential fluctuations and diffusion‐pathway hysteresis induced by rainfall pulses, (ii) the temperature sensitivity of microbial respiration and gas diffusivity, and (iii) thermal–hydraulic feedbacks across terminal (surface‐groundwater) boundaries, which together govern CO_2_ production, migration, and retention under non‐steady‐state field conditions.

The soil column (0–200 cm) was discretized into six functional layers (0–10, 10–30, 30–50, 50–100, 100–150, 150–200 cm) reflecting observed gradients in structure, bulk density (Figure S2a) and carbon content (Figure S3). Depth‐dependent hydraulic, thermal, and biogeochemical parameters (Tables S1–S5) were derived from laboratory and field measurements, incorporating both matrix and preferential flow domains. The model domain comprised 301 computational nodes with refined near‐surface resolution to capture steep gradients in moisture, temperature, and CO_2_.

Boundary conditions included daily meteorological forcing at the surface and free drainage at the bottom to reflect deep internal water redistribution (Figure 2a). Water and heat boundaries were updated daily, matching field temporal resolution. Simulations spanned a full annual cycle (August 2024–July 2025) using adaptive time steps (≤ 0.005 days; ~7.2 min) to ensure stability during rapid infiltration or thermal events (Šimůnek et al. 2008, 2006).

**FIGURE 2:**
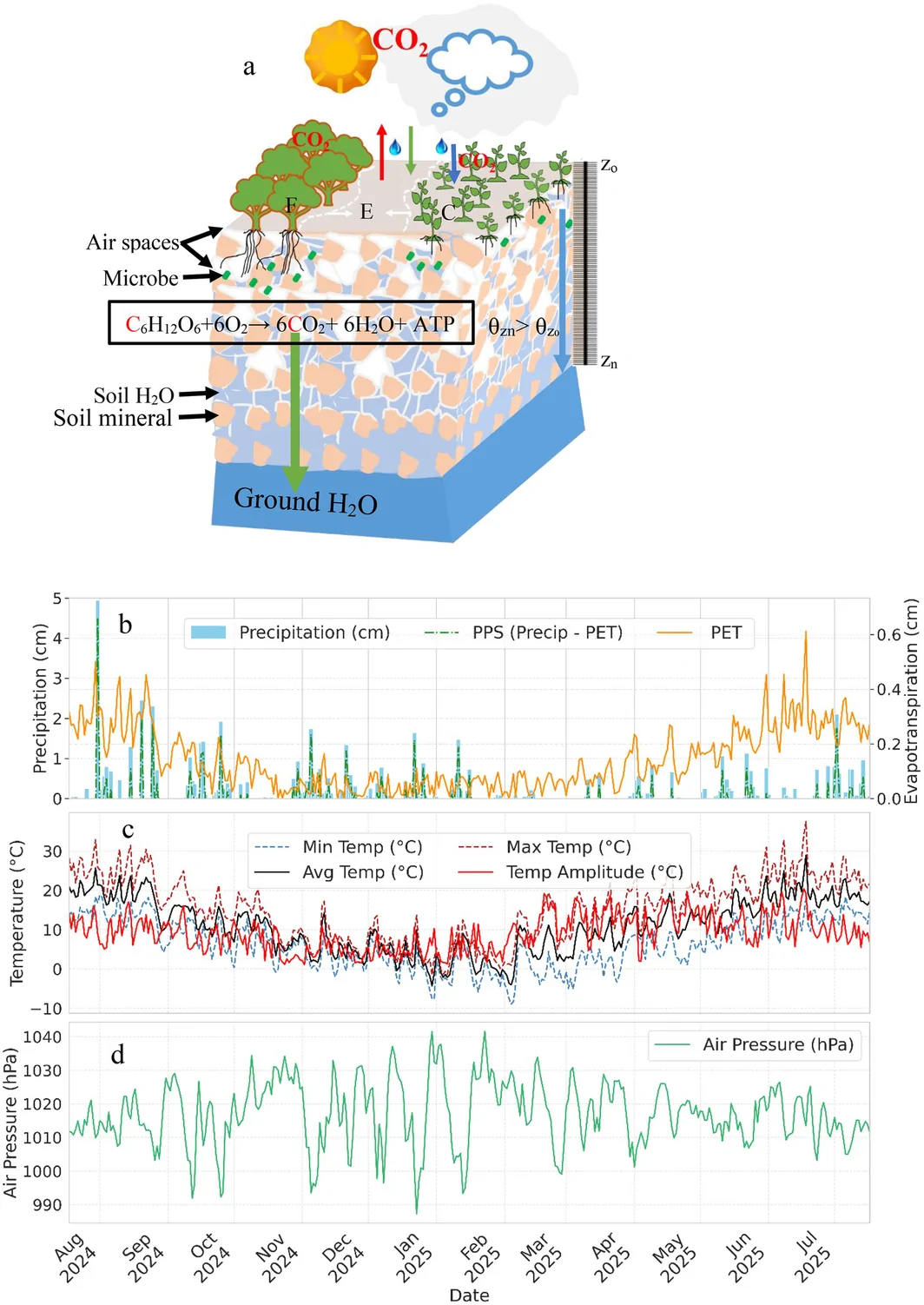
DeepCO_2_ framework and seasonal hydroclimatic forcing. (a) Conceptual diagram of the DeepCO_2_ framework linking soil‐atmosphere processes that regulate water, carbon and energy exchange across land uses (forest, F; cropland, C; edge, E). Precipitation infiltrates (blue arrows) and redistributes through soil layers (z_0_−z_n_), establishing hydrostatic gradients (*θ*_zn_ > *θ*_z_), while evapotranspiration (red arrow) couples land use with atmospheric demand. Microbial and root respiration (C_6_H_12_O_6_ + 6O_2_ → 6CO_2_ + 6H_2_O + ATP) oxidizes organic carbon to CO_2_ that diffuses upward (red arrow) or is advected downward (green arrows). Differences in organic carbon supply, moisture and thermal retention, and root‐microbe interactions regulate CO_2_ dynamics across depth and season. (b–d) Seasonal variation in hydroclimatic drivers at Fuhrberger Feld (August 2024–July 2025). (b) Precipitation (bars), potential precipitation surplus (PPS, dashed green line) and Penman‐Monteith evapotranspiration (orange line). (c) Daily minimum, mean and maximum air temperature and diurnal amplitude. (d) Atmospheric pressure dynamics. Soil physical, thermal, hydraulic and biological parameters used for model calibration are provided in Tables S1–S5. Depth distributions of bulk density, soil organic carbon (SOC) stocks, microbial biomass carbon (MBC) stocks, and root biomass are provided in Figures S2–S5.

#### 2.6.1 Rainfall‐Bound Water Flow Sub‐Model Within the DeepCO_2_ Framework

Water flow within the unsaturated zone was numerically solved using the Richards equation, coupling volumetric water content (*θ*), matric pressure head (*h*), and hydraulic conductivity (*K*). Soil hydraulic properties followed the van Genuchten (1980) retention model and the Mualem (1976) conductivity model:

(1) $$\theta \left(h\right)=S_{e}\left(h\right)\;\left[\left(\theta_{s}-\theta_{r}\right)+\theta_{r}\right]$$

(2) $$K\left(h\right)=K_{s}S_{e}{\left(h\right)}^{0.5}{\left[1-{\left(1-{S_{e}}^{\frac{1}{m}}\right)}^{m}\right]}^{2}$$

where the degree of water content saturation, $S_{e}\left(h\right)={\left[\frac{1}{1+{\left(\alpha \left|h\right|\right)}^{n}}\right]}^{m}$, *θ*_*r*_ and *θ*_*s*_ denote the residual and saturated soil water content (cm^3^ cm ^−3^), respectively, and *n* (−), *m* (−) and *α* (cm^−1^) are empirical parameters. In this study, *m* was constrained as 1−1/*n*.

Hydraulic parameters were independently assigned to each of the six main soil layers across all nine experimental sites (Table S2), accounting for pore size distribution and structural effects. Transient simulations were driven by daily rainfall input at the soil surface, coupled with free drainage at the lower boundary, allowing the model to capture dynamic infiltration, evapotranspiration, and deep redistribution patterns with high spatial and temporal fidelity (Figure 2b). Daily forcing, rather than monthly averages, was critical to resolving rapid wetting‐drying cycles that drive short‐term CO_2_ pulses in seasonally unsaturated soils.

#### 2.6.2 Heat Transport Sub‐Model Within the DeepCO_2_ Framework

Heat transport in the soil profile was modeled using the coupled conduction‐advection equation of Sophocleous (1979):

(3) $$C\frac{\partial T}{\partial t}=\frac{\partial }{\partial z}\left\{\lambda \left(\theta_{w}\right)\frac{\partial T}{\partial z}\right\}-C_{w}\frac{\partial q_{w}T}{\partial z}$$

*C* and *C*_*w*_ are the volumetric heat capacities (J m^−3^ K^−1^) of the soil matrix and water phase, respectively, *λ*(*θ*_*w*_ *)* is the apparent thermal conductivity (W m^−1^ K^−1^), dependent on water content and flow velocity, *q*_*w*_ is the Darcian water flux (m s^−1^) and *T* is soil temperature (K).

The volumetric heat capacities were computed from bulk density and gravimetric heat capacity values, differentiating between mineral and organic fractions to reflect their distinct thermal storage capacities (Tables S3 and S4). Apparent thermal conductivity was modeled as a composite function incorporating both static conduction and advective heat transport (de Marsily 1986):

(4) $$\lambda \left(\theta \right)=\lambda_{0}\left(\theta \right)+B_{t}C_{w}|q_{w}|$$

Here, *λ*_0_(θ) is the baseline thermal conductivity as a function of water content, and *B*_*t*_ denotes thermal dispersivity (cm), representing enhanced heat transfer by advective water flow. The static component *λ*_*0*_ (*θ*) (Wcm^−1^ °C) was parameterized following Chung and Horton (1987):

(5) $$\lambda_{0}\left(\theta \right)=b_{1}+b_{2}\theta +b_{3}\theta^{0.5}$$

where *b*_1_, *b*_2_, and *b*_3_ are empirically calibrated coefficients specific to each soil layer (Tables S3 and S4), reflecting the soil texture and organic carbon content. The influence of soil organic matter (SOM) on thermal properties was scaled using the Van Bemmelen factor (1.724), assuming organic matter contains approximately 58% carbon. The SOM fraction was computed as follows:

(6) $$\mathrm{SOM}\;\text{fraction}=\frac{\bar{\mathrm{\rho b}}}{\mathrm{\rho s}}\cdot \frac{1.724\times \mathrm{SOC}\%}{100}$$

where $\bar{\mathrm{\rho b}}$ (g cm^−3^) is the mean bulk density obtained from three replicate soil cores (Figure S2), SOC (%) is the measured soil organic carbon (Figure S3), and *ρs* = 1.3 g cm^−3^ is the reference particle density of organic matter. The volume fraction of the solid mineral phase (*SF*) was calculated as:

(7) $$\mathrm{SF}=\frac{\bar{\mathrm{\rho b}}}{\mathrm{\rho p}}$$

where *ρ*_*p*_ = 2.65 g cm^−3^ is the particle density of mineral soil, providing a dimensionless measure of the proportion of soil volume occupied by mineral solids, directly influencing thermal inertia.

Upper boundary soil temperature was imposed via a sinusoidal forcing function, capturing both seasonal and diurnal oscillations (Šimůnek and Suarez 1993; Kirkham and Powers 1972):

(8) $$T=T_{0}+A\;\sin\frac{2\mathrm{\pi t}}{p}+\frac{7\pi }{12}$$

*T*_*0*_ is the observed daily‐corrected monthly mean surface temperature, *A* is the monthly mean diurnal amplitude (computed as the average difference between daily max and min air temperatures) accounting for the typical intra‐day oscillations at the imposed soil surface boundary (Figure 2b), *p* is the period of one diurnal cycle (1 day) of one sine wave of temperature. The phase shift (7π/12) accounts for peak surface temperature typically occurring around 13:00 (Šimůnek and Suarez 1993). This formulation captures rapid daytime heating and nocturnal cooling essential for resolving short‐term feedbacks between temperature and CO_2_ fluxes during sensitive phenological stages.

For days without direct temperature measurements, the surface temperature *T*_0i_^top^ was constructed using meteorological air temperatures at the Fuhrberger Feld station by scaling air temperature on missing days against days with known soil surface temperatures, thus maintaining physical consistency across the simulation period (Figure 2c).

(9) $$T_{0_{i}}^{\mathrm{top}}=\frac{T_{\alpha_{i}}}{T_{\beta }}\times T_{m}^{\mathrm{top}}$$

where *Tα*_*i*_ is the air temperature on the missing day *i*, *T*_*β*_ is the air temperature corresponding to a day with known soil surface temperature, and *T* ^*top*^_*m*_ is the measured soil surface temperature for that reference day. This correction ensured the thermal boundary remained physically consistent across the entire simulation period.

At the lower boundary, capillary fringe‐unsaturated zone interface temperature was modeled to reflect strong thermal inertia. For missing days, the bottom boundary temperature *T*_0i_^bot^ was reconstructed using an empirically derived damping factor of 3.5, reflecting attenuation of short‐term thermal fluctuations:

(10) $$T_{0_{i}}^{\mathrm{bot}}=T_{\alpha_{i}}\times \exp\left(-3.5\right)+T_{m}^{\mathrm{bot}}$$

where *Tα*_*i*_ is the air temperature on the missing day *i*, and *T* ^bot^_*m*_ is the measured capillary fringe temperature on the reference day. This approach preserves the slow‐responding thermal signal at depth, which plays a pivotal role in modulating deep CO_2_ transport and seasonal production dynamics.

#### 2.6.3 Soil CO_2_ Production and Transport Sub‐Model Within the DeepCO_2_ Framework

The soil CO_2_ dynamics submodel incorporated depth‐resolved production rates, gas diffusion, and advection mechanisms within the DeepCO_2_ framework. Simulations of CO_2_ balance in soil profiles under varying land uses deployed the SoilCO_2_ module, which explicitly accounts for the primary biological CO_2_ production processes (root respiration and microbial carbon mineralization), modulated by environmental variables (soil moisture, temperature, osmotic conditions, and CO_2_ concentration) (Šimůnek and Suarez 1993; Suarez and Šimůnek 1993). The total CO_2_ production rate *S*_*T*_ (cm^3^cm^−2^d^−1^) across the soil profile of length *L* is calculated as the integral sum of microbial (*γ*_*m*_) and plant root (*γ*_*p*_) respiration terms, scaled by multiple depth‐ and condition‐specific response functions:

(11) $$\begin{array}{c} S_{T}=\int_{0}^{L}S\;\mathrm{dz}\;=\;\gamma_{m}^{O}\;f_{m}\left(t\right)\int_{0}^{L}f_{m}\left(z\right)f_{m}\left(h\right)f_{m}\left(h_{\vartheta }\right)f_{m}\left(T\right)f_{m}\left(c_{\alpha }\right)\;\mathrm{dz}\\ +\gamma_{p}^{O}f_{p}\left(t\right)\int_{0}^{L}f_{p}\left(z\right)f_{p}\left(h\right)f_{p}\left(h_{\vartheta }\right)f_{p}\left(T\right)f_{p}\left(c_{\alpha }\right)\;\mathrm{dz} \end{array}$$

where *γ*_*m*_^*o*^ and *γ*_*p*_^*o*^ are the optimal (~20°C, −1020 hPa, optimal solute and gas concentrations) potential CO_2_ production rates (cm^3^ cm^−2^ d^−1^) for microorganisms and plant roots, respectively, *f*(*t*) represents temporal variation, capturing diurnal and seasonal dynamics in microbial and root activity, *f*(*z*) captures the vertical distribution of CO_2_ accumulation, microbial and root activities with depth, *f*(*h*) accounts for soil water stress, reducing respiration under saturated (increasingly‐limited oxygen) or dry (drought) conditions, *f*(*h*_*ϑ*_) resolves osmotic stress, reducing respiration due to solute‐induced stresses, *f*(*T*) modulates soil temperature‐controlled respiration, *f*(*c*_*α*_) adjusts for CO_2_‐enrichment feedback inhibition of root respiration at high CO2 concentrations (Šimůnek and Suarez 1993; Suarez and Šimůnek 1993).

The dependence of CO_2_ production on CO_2_ concentration, influenced by thermal and hydraulic gradients and the depth decay of root and microbial activity, was described using a Michaelis–Menten equation (Glinski and Stępniewski 1985).

(12) $$q=\frac{q_{\max}}{1+\left(K_{Mc_{O_{2}}}\right)}$$

here, *q* is the CO_2_ production rate and *q*_max_ is the maximum CO_2_ production rate (cm^3^ cm^−3^ d^−1^). *K*_*M*_ is the Michaelis constant (cm^3^ cm^−3^), that is, the CO_2_ concentration *c*CO_2_ at which CO_2_ production is reduced by half from its optimal value.

The depth dependence of the production term *f*_*m*_(*z*) was solved using the exponential distribution function (*f*) based on the vertical distribution of soil organic matter, root, and microbial biomass (Figures S3–S5) (Glinski and Stępniewski 1985).

(13) $$f=a\;\exp\left\{-a\left(L-z\right)\right\}$$

where *L* is the soil surface coordinate and *a* is an empirical decay constant (cm^−1^) (Table S5).

CO_2_ transport through the porous medium was described by solving the mass balance equation, based on Fick's law to represent diffusive fluxes combined with hydrodynamic dispersion (Šimůnek and Suarez 1993):

(14) $$\frac{\partial C_{T}}{\partial t}=-\frac{\partial }{\partial z}\left(J_{\mathrm{da}}+J_{\mathrm{dw}}+J_{\mathrm{ca}}+J_{\mathrm{cw}}\right)-{\mathrm{Qc}}_{w}+S$$

where *C*_*T*_ is the total CO_2_ concentration, *J* terms represent advective and diffusive fluxes in gaseous and aqueous phases, *Qc*_*w*_ is the advective transport in the liquid phase, and *S* is the CO_2_ source term from microbial and root respiration (Equation 11). The diffusion coefficients in the gas and aqueous phases, *D*_*a*_ and *D*_*w*_, respectively, were computed as follows:

(15) $$D_{w}=D_{\mathrm{ws}}\tau_{w}+\lambda_{w}\left|\frac{q_{w}}{\theta_{w}}\right|$$

(16) $$D_{a}=D_{\mathrm{as}}\tau_{a}$$

where *D*_*ws*_ and *D*_*as*_ are the temperature‐dependent molecular diffusion coefficients (cm^2^ d^−1^) of CO_2_ in water and air, *τ*_w_ and *τ*_a_ are tortuosity factors (cm cm^−1^) accounting for the porous medium (aqueous and gaseous, respectively) geometry and phase connectivity, and *λ*_*w*_ is the dispersivity in the aqueous phase. The tortuosity factors were calculated following Millington and Quirk (1961), inversely related to porosity and proportional to phase saturation. Due to the dominance of diffusion over mechanical dispersion in the gaseous phase (except air velocity is very high under extreme turbulence such as cyclones) under field conditions, the model assumes irrelevant mechanical dispersive gas transport (Šimůnek and Suarez 1993).

Because HYDRUS does not simultaneously couple water and air movement, air fluxes *q*_*a*_ were assumed negligible at the lower boundary, such that volumetric changes in water content *q*_*w*_ */θ*_*w*_ are compensated by gas volume changes in the pore space under variably saturated moisture regimes, consistent with most field scenarios (Šimůnek and Suarez 1993; Suarez and Šimůnek 1993). At the soil surface, CO_2_ concentration *ca*_*0*_ was set equal to the atmospheric equilibrium value (~350–850 μmol mol^−1^), with measured surface CO_2_ concentrations from chamber experiments across nine field sites informing spatial variability. A first‐type Dirichlet boundary condition was imposed at the soil‐air interface, where effective CO_2_ diffusion coefficients incorporated the combined soil air and water flux effects, which emerge from the coupled solution of water flow and gas transport equations (Šimůnek and Suarez 1993; Suarez and Šimůnek 1993).

This integrated CO_2_ production and transport submodels provide a mechanistic, depth‐ and time‐resolved representation of soil CO_2_ dynamics, enabling quantification of biological activity and gas exchange processes under varying environmental and land use conditions (Figure 2).

## 3 Results

### 3.1 Climate‐Driven Hydraulic and Thermal Signatures Across Land Uses

Strong vertical and seasonal structuring was observed in water‐filled porosity (WFP) across cropland, forest, and edge profiles from August 2024 to July 2025 (Figure 3a–c), increasing with depth and toward winter between ~15% to 78%, reflecting rainfall‐evapotranspiration dynamics. Across land uses, the rhizosphere (≤ 100 cm) generally maintained oxic/field‐capacity conditions (WFP ~40%–70%), reflecting land‐use‐specific hydraulic properties, root water uptake, and atmospheric demand. In contrast, deeper layers (> 100 cm) were dominated by gravitational internal water redistribution and remained wetter (WFP > 80%; Figure 3a–c).

**FIGURE 3:**
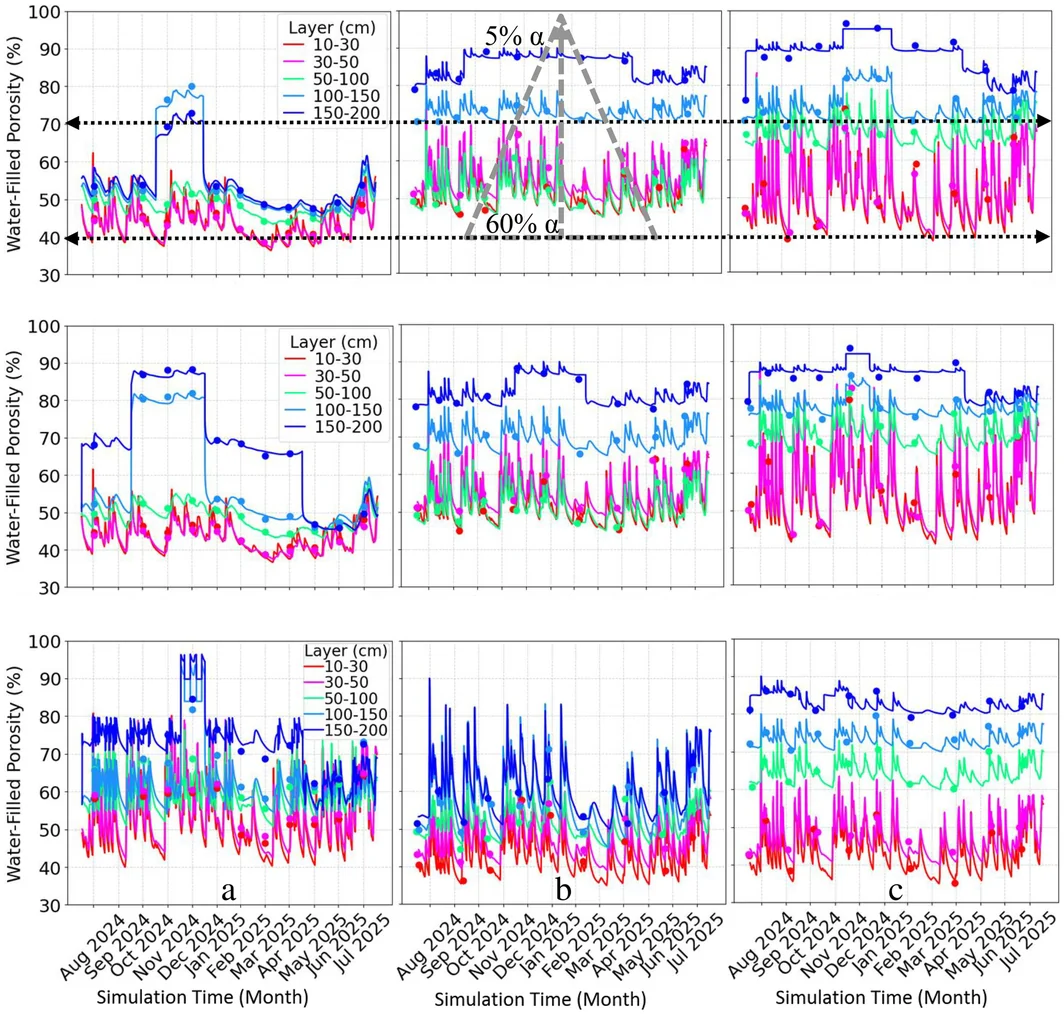
Average depth‐ and season‐resolved water‐filled porosity (WFP, %) across cropland (a), edge (b), and forest (c) profiles from August 2024 to July 2025. Air‐filled porosity (α) generally declined with depth (≈60% to 5%; arrowed triangle) and toward winter. Horizontal arrows indicate the aeration‐optimal range (WFP ~40%–70%), beyond  which increasing water saturation (> 80% WFP) indicates O_2_‐deficient conditions in deeper layers. Each row represents one of the three triads of land‐cover classes. Moisture content profiles are reported in Figure S6.

Soil temperature profiles showed a concave, depth‐ and land‐use‐specific stratification (Figure 4a–c), with strong diurnal and seasonal amplitudes (5°C–13°C) in the soil‐atmosphere interface (0–10 cm) that were progressively muted at depth (150–200 cm). The largest soil‐atmosphere interface variability 31°C–35°C in summer (peaks > 38°C) and −4°C in winter was observed in cropland and forest‐cropland edge (Figure 4a,b), while subsurface layers fluctuated moderately (50–200 cm: 5°C–28°C). Forests remained thermally stable, with surface means 25°C–29°C and deep 8°C–18°C (Figure 4c), reflecting canopy shading, litter insulation and moisture‐mediated thermal buffering.

**FIGURE 4:**
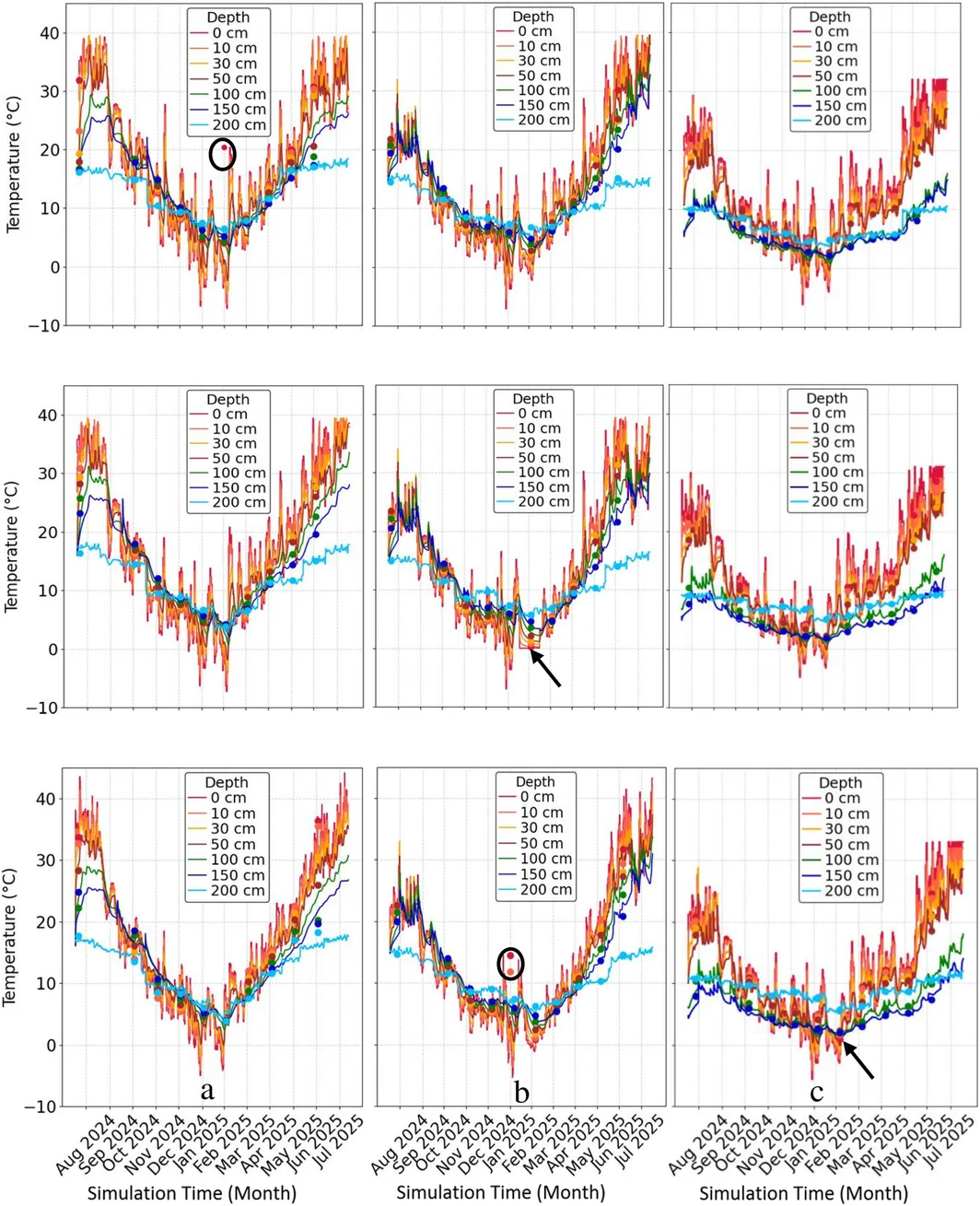
Depth‐ and season‐resolved soil temperature profiles across cropland (a), edge (b), and forest (c) systems from August 2024 to July 2025. Agreement between measured (points) and simulated (curves) values confirms the DeepCO_2_ framework's capacity to reproduce land‐use‐specific thermal dynamics and amplitude contrasts (maxima = circles; minima = arrows). Each row represents one of the three triads of land‐cover classes.

### 3.2 Daily to Seasonal Soil CO_2_ Dynamics Reveal a Production‐Transport Imbalance Across Land Uses

Simulated soil CO_2_ production (August 2024–July 2025) varied strongly with land use, depth, and season (Figure 5). Production rate peaked in the upper 30 cm (~4–6 × 10^4^ μmol mol^−1^ d^−1^) and declined exponentially with depth (Figure 5a–c), rarely exceeding 10^3^–10^4^ μmol mol^−1^ d^−1^ below 50 cm and with vertical patterns regulated by root and organic matter distribution (Figures S3 and S4). During warm periods, root respiration and microbial SOC decomposition extended to 50–80 cm in cropland and edge soils, and to ~100 cm in forests, tracking deepening thermal and aeration fronts (Figures 3 and 4), whereas winter dormancy and cold inhibition (December–February) brought CO_2_ production to minimum levels (< 10^3^ μmol mol^−1^ d^−1^).

**FIGURE 5:**
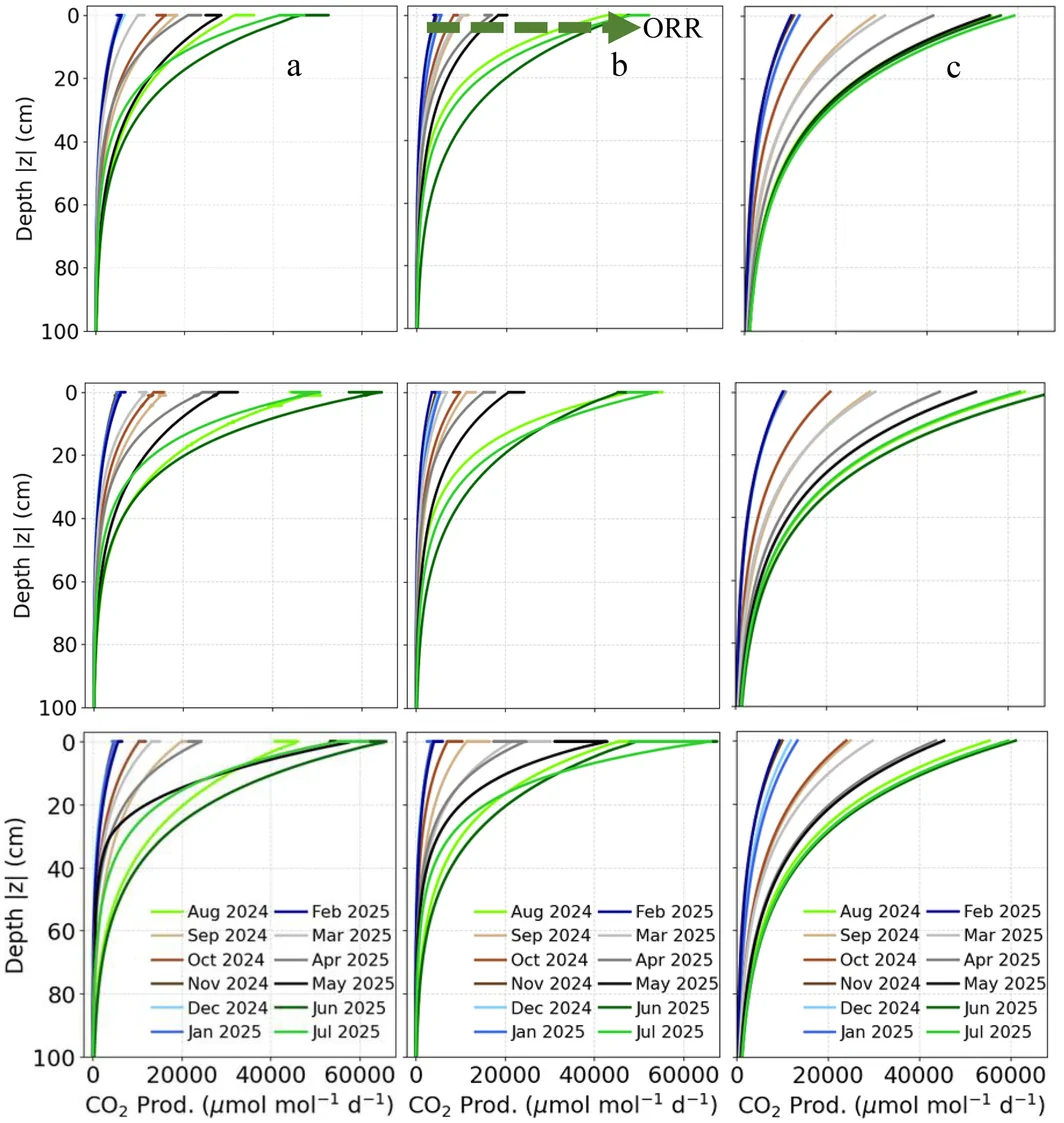
Simulated soil CO_2_ production (μmol mol^−1^  d^−1^; August 2024–July 2025) across croplands (a), edges (b), and forests (c), showing optimal respiration rate (ORR, green arrow) toward summer and exponential decay with depth. Each row represents one of the three triads of land‐cover classes.

Soil CO_2_ concentration dynamics (Figure 6a–c) displayed pronounced diel and seasonal variability, with shallow layers (10–50 cm) driving the largest diurnal oscillations; daytime peaks of ~10,000 to 60,000 ppm in spring and summer, and nocturnal and winter minima near atmospheric equilibrium (~350–750 ppm). Diurnal variations were muted in deep layers (100–200 cm), where CO_2_ concentration reached ~12–318 times atmospheric (i.e., 0.1–1 × 10^5^ μmol mol^−1^; Figure 6), far exceeding (~9–17 times) concentrations expected from in situ production alone (Figure 5), with the greatest amplification (2–2.26 times) in forests (Figure 6c), reflecting a clear production‐transport imbalance.

**FIGURE 6:**
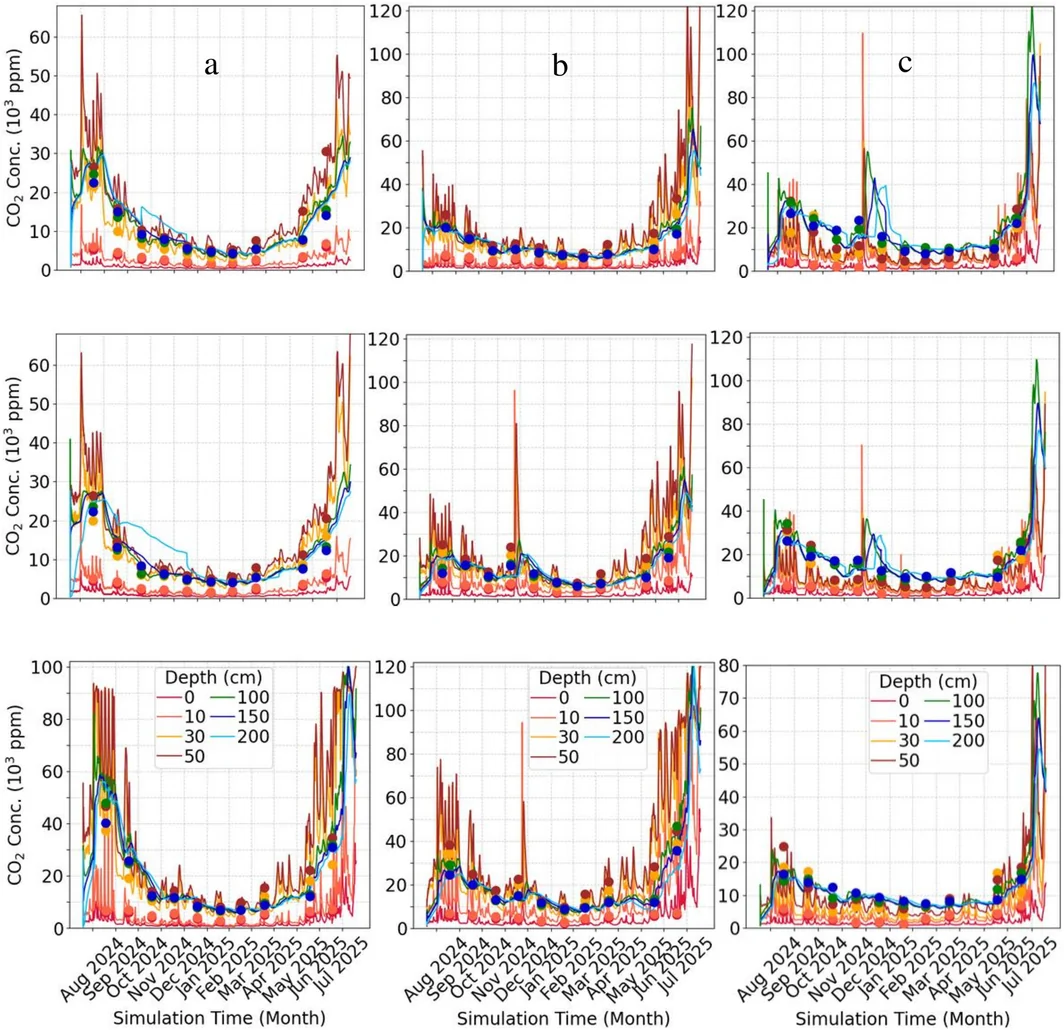
Depth‐ and season‐resolved soil CO_2_ concentrations (ppm) across cropland (a), edge (b), and forest (c) systems from August 2024 to July 2025 (simulation: Lines, measurement: Points). Each row represents one of the three triads of land‐cover classes. Blue scatter represents measured average CO_2_ concentrations at 150 and 200 cm depth.

## 4 Discussion

### 4.1 Land‐Cover‐Associated Differences in Vertical Soil CO_2_ Production Under Climate Forcing

Distinct vertical and seasonal structuring of soil CO_2_ production emerges across land‐cover types, with the highest production concentrated in the upper ~30 cm and progressively lower production with increasing depth (Figure 5). These topsoil CO_2_ production hotspots coincide with concentrated organic matter, denser rooting, and comparatively favorable temperature and moisture conditions sustaining > 90% of total respiration (Glinski and Stępniewski 1985; Campbell 1985). With depth, declining organic carbon availability (Figure S3) reduces substrate availability for microbial activity (Figure S5), while decreasing root biomass (Figure S4) reduces the contribution of root respiration. At the same time, increasing suboptimal moisture‐temperature regimes (Figures 3 and 4) progressively constrain microbial and root metabolism, establishing increasingly restrictive environmental conditions for biological activity and subsoil carbon turnover (Giardina et al. 2014; Šimůnek and Suarez 1993; Suarez and Šimůnek 1993).

Within the temperate thermal envelope (−4°C to 40°C; Figure 4), seasonal radiative forcing during spring–summer optimizes soil temperature (20°C–35°C) and moisture (40%–70% WFP; Figure 3), accelerating enzymatic reactions, substrate oxidation, and carbon turnover in both autotrophic and heterotrophic systems (Holden and Fierer 2005; Reichstein and Beer 2008; Tang et al. 2022) and producing peak CO_2_ fluxes (~4 × 10^4^–6 × 10^4^ μmol mol^−1^ d^−1^; Figure 5). Beyond thermal and moisture optima, enzyme instability and energy imbalance (driven by coupled anoxia and cold‐stress inhibition of electron transfer and ATP synthesis) increase, respiration rates decline, and energy yield per unit substrate decreases (Šimůnek and Suarez 1993; Suarez and Šimůnek 1993; Glinski and Stępniewski 1985), shifting metabolism away from optimal CO_2_ production, as during winter dormancy when cold spells (−4°C to 15°C) depress CO_2_ production by up to ~150‐fold relative to summer (Figure 5). Oversaturation (> 80% WFP) restricts O_2_ diffusion, while dry spells (< 30% WFP) limit metabolic activity and substrate solubility (Figure 5). Because aeration declines hydrostatically with depth (~62% to 5%; Figure 3) (Cornelis et al. 2019; Hillel 2003; Mualem 1976), O_2_ deficiency intensifies (from near‐atmospheric levels ~20.5 kPa to < 2–5 kPa), approaching hypoxic thresholds for aerobic metabolism, respiration efficiency decreases sharply (up to 92%; Figure 5), and CO_2_ production becomes increasingly constrained below 30 cm (Chen et al. 2021).

Land‐cover type influences these environmental controls. In croplands and edges, sparse canopy cover amplifies surface warming (22°C–40°C; Figure 4a,b) and evaporation (Chung and Horton 1987; Sophocleous 1979), promoting deep optimal thermal (~24°C–27°C) and aeration fronts (50–100 cm; WFP = 40%–60%, Figures 3a,b and 4a,b) that support active SOC‐derived CO_2_ production in root‐diminished subsoils (~30–80 cm; Figure S4) during spring–summer periods (Figure 5a,b) (Meyer et al. 2018; van ‘t Hoff 1896; Arrhenius 1889). In forests, elevated organic carbon availability (Figure S3) and denser rooting (Figure S4) sustain pronounced topsoil CO_2_ production (Figure 5c), but canopy‐litter insulation dampens thermal gradients (15°C–18°C), confining microbial activity to relatively warmer shallow layers (Figure 4) (Bogati and Walczak 2022). The sharp decline in organic matter with depth (Figure S3), superimposed on temperature limitations (Figure 4) to forest subsoil microbial carbon cycling (Figure S5), results in the contribution of SOC (Figure S3) to CO_2_ production becoming negligible below 30 cm, whereas deeper roots (Figure S4) become the dominant contributor to CO_2_ production extending the production fronts to ~100 cm in forest subsoils (Figure 5c) (Billings et al. 2024, 2018; Wordell‐Dietrich et al. 2020).

This contrast highlights vegetation's dual role under climate warming, enhancing rhizospheric carbon enrichment while preserving subsoil SOC pools where suboptimal temperatures constrain microbial activity and SOC‐derived CO_2_ production (Dong et al. 2026; Wordell‐Dietrich et al. 2020; Glinski and Stępniewski 1985; Campbell 1985; de Vries 1963).

These findings demonstrate that land‐use type does not simply influence the magnitude of soil CO_2_ production, but is associated with differences in its vertical distribution and apparent climate sensitivity. By extending optimal temperatures to depths approaching 1 m, where microbial biomass can be 87% ± 26% greater in cropland than forest and 200% ± 47% greater in forest‐cropland edges than forest (Figure S5), cropland and forest‐cropland edge systems may increase the vulnerability of subsoil SOC to warming, potentially enhancing microbial processing, especially below ~30 cm. This depth‐dependent persistence of microbial activity and expansion of SOC vulnerability suggests a potential mechanism through which differences in land‐cover may influence the climate sensitivity of soil organic carbon. Because most Earth system models represent soil respiration as a vertically integrated, surface‐weighted temperature response (Tian et al. 2010), they may not fully capture this vertical restructuring of bioactivity. As a result, current carbon‐climate projections could underestimate the potential contribution of deep SOC to soil‐atmosphere feedbacks under warming and contrasting land‐cover conditions.

Importantly, because the study sites were not established through a controlled land‐use conversion experiment, differences in inherent site characteristics that may have influenced historical land‐use decisions (Hardie et al. 2009; Springob et al. 2002) could also contribute to the observed patterns. We therefore interpret these results as associations between current land cover and the environmental controls on vertical CO_2_ production, rather than as evidence that land‐use change alone caused the observed differences. Moreover, under other climatic and hydrological settings, particularly in (semi) arid systems with deeper water tables, deeper rooting zones, and warmer subsoils favorable for microbial and root activity in forests may extend well below the 1 m depth observed here (Figure 5c) (e.g., Rowan et al. 2025; Billings et al. 2018). Such conditions could sustain substantial subsoil CO_2_ production below 1 m, and therefore the 1 m depth observed in this study should not be interpreted as a universal metabolic boundary limiting root respiration or subsoil carbon turnover.

### 4.2 Land‐Use‐Dependent Subsoil CO_2_ Enrichment Defines Boundary‐Driven Soil‐Atmosphere Exchange of Respired CO_2_

Across all land uses, CO_2_ concentrations measured at 0–10 cm closely match model simulation, reflecting near‐atmospheric signal at the soil‐atmosphere interface (Figure 6). Below 10 cm, and particularly within the upper 30 cm, however, both measured and modeled CO_2_ concentrations consistently exceed concentrations at the soil‐atmosphere boundary, indicating a persistent production surplus and a dual pathway through which respired CO_2_ can either be exchanged with the atmosphere or redistributed into deeper soil layers (Figure 6) (Tang et al. 2022; Šimůnek and Suarez 1993; Suarez and Šimůnek 1993). As such, the upper 30 cm constitutes a biophysical gateway through which land‐cover‐associated differences in CO_2_ production and transport may influence the balance between atmospheric exchange and downward CO_2_ redistribution (Raich and Tufekciogul 2000; Guggenberger et al. 2020; Leiber‐Sauheitl et al. 2014).

The 30–100 cm layer functions as a mixed production‐redistribution zone characterized by reduced in situ respiration (10%–25%; Figure 5) but dominated by advective CO_2_ inputs from shallower layers that exceed concentrations attributable to local production by 2–17.5‐fold (Figure 6), revealing a clear production‐transport imbalance in which influxes from more bioactive topsoil surpass in situ respiration (Moldrup et al. 2004, 2003; Welles et al. 2001; Millington and Quirk 1961). This apparent sink capacity is 2–2.3‐fold greater in forests than in cropland and edge soils (Figure 6). In forests, abundant tortuous flow paths associated with fissures, cracks, and root channels strengthen macroporosity (ϕ–θ at −30 cm H_2_O) and promote longitudinal dispersivity and advective coupling between shallow (≤ 50 cm) and deeper (50–200 cm) layers (Figure 7c) (Oh et al. 2005; Iversen, Moldrup, et al. 2001; Iversen, Schjønning, et al. 2001), maintaining effective gas diffusivity (De = 0.6–0.8 × 10^−6^ m^2^ s^−1^) and driving CO_2_ accumulation in the 50–100 cm layer up to 10–17.5 times greater than can be inferred from local production alone and in the 100–200 cm layer to 0.12–1.20 × 10^5^ μmol mol^−1^ (12,000–120,000 ppm), equivalent to ~20–242 times atmospheric concentrations (Figures 5c and 6c). This variability likely reflects the balance between production and transport constraints, with seasonal CO_2_ exchange enhanced during spring–summer as production (Figure 5) increases by an average of 115‐fold compared with winter and temperature‐porosity‐dependent diffusivity (Figure 7) favors deep CO_2_ loading in concentrations ~12–22‐fold greater than in winter, while also strengthening the surface signal by ~1.23–2.22‐fold. By contrast, elevated thermal gradients (24°C–35°C; Figure 4a,b) and reduced macroporosity (~27%–30% in cropland; ~19%–21% in edge) in cropland and edge soils weaken gas transport pathways, disrupting surface‐subsurface continuity and transforming the shallow layer into an efficient outgassing zone overlying a transport‐limited domain (Figure 7a,b) (Xiong et al. 2025; Chen 2019; Koehler et al. 2010; Iversen, Moldrup, et al. 2001; Iversen, Schjønning, et al. 2001). Consequently, deep CO_2_ loading is reduced by ~50%–57% relative to forest soils but still exceeds in situ production by 4–13 times (Figures 5a,b and 6a,b), reflecting weakened advective coupling and diminished emission‐mitigation capacity.

**FIGURE 7:**
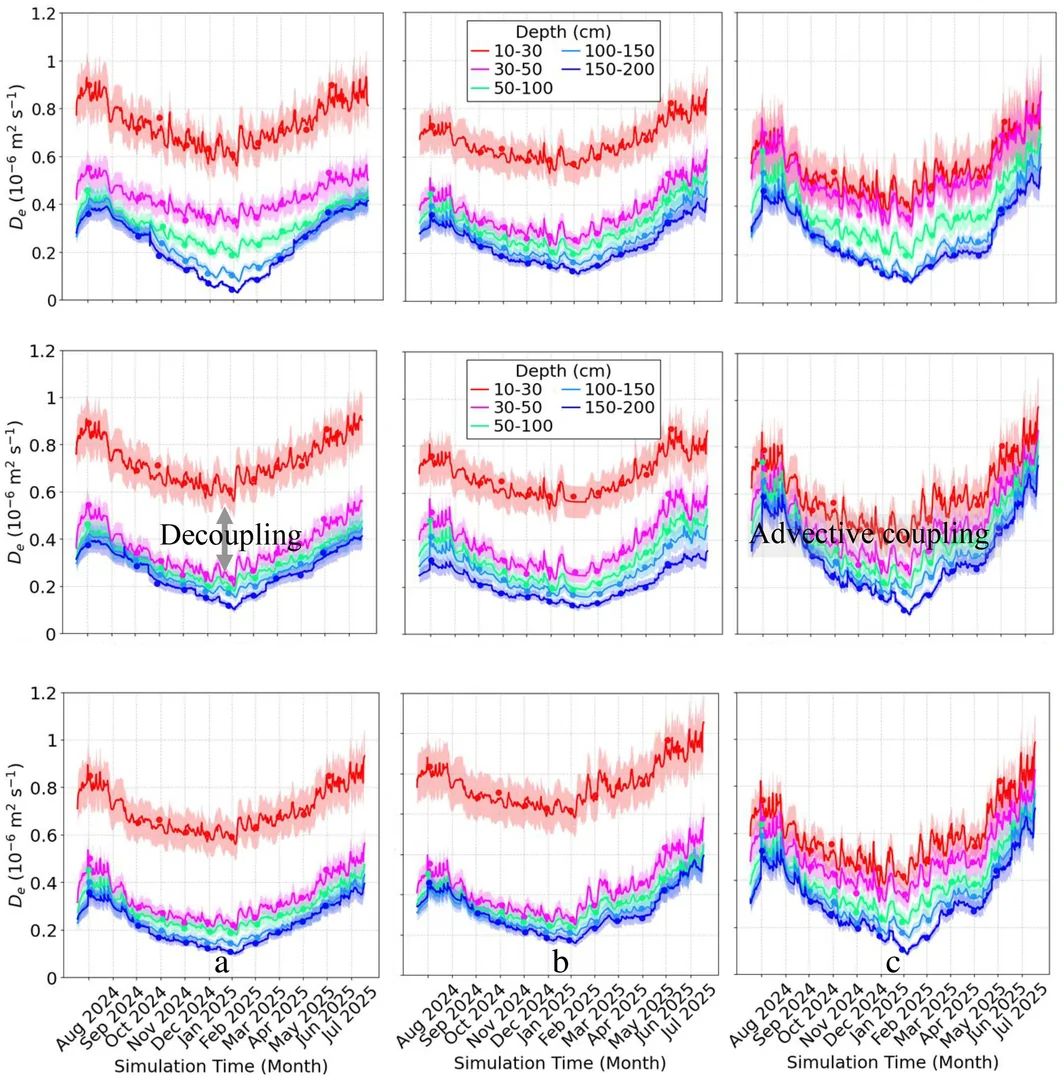
Depth‐ and season‐resolved effective diffusion coefficient (D_e_; ×10^−6^ m^2^ s^−1^) across cropland (a), edge (b), and forest (c) profiles from August 2024 to July 2025. Elevated advective decoupling (double arrow) between shallow (≤ 30 cm) and deeper layers in croplands and edges indicates weakened surface‐subsurface connectivity. Each row represents one of the three triads of land‐cover classes.

These contrasts demonstrate that land‐cover and management differences among forests, croplands, and forest‐cropland edges/fragmented landscapes are associated with effective “leak points” that can amplify soil‐atmosphere CO_2_ exchange while reducing the retention of respired CO_2_ in subsoil domains (Garvey et al. 2022; Murcia 1995). By governing whether CO_2_ produced in the most bioactive layers is rapidly vented or progressively transferred downward, under the studied temperate and hydrological conditions, the 30–100 cm zone defines a functional transition between active CO_2_ production and transport‐dominated redistribution (Figure 5), regulating emissions and deep carbon loading. Structural degradation through compaction, reduced porosity, or over‐ventilation impairs this buffering and diverts CO_2_ toward the atmosphere, as observed in cropland and edge systems (Gui et al. 2023; Soane and van Ouwerkerk 1994), whereas maintaining pore connectivity, root penetration, and water balance strengthens subsurface sink capacity (Paul et al. 2004; Hillel 2003).

Below 100 cm, increasingly limited oxygen (Figure 3) and suboptimal temperatures (Figure 4) suppress in situ respiration (Figure 5), and CO_2_ dynamics are dominated by transport‐limited accumulation (Figure 6), defining the terminal node of soil CO_2_ cycling and a potential deep CO_2_ storage pool. Thus, even when moisture exceeds the optimum range for subsoil respiration because of oversaturation (Figure 3), restricted CO_2_ diffusion (Figure 7) can allow biologically produced CO_2_ (Figure 5) to accumulate in deeper soil domains (Figure 6) (Hasenmueller et al. 2015; Oh et al. 2005). Importantly, the fate of CO_2_ in this zone may extend beyond temporary gaseous storage. High CO_2_ concentrations (Figure 6) in “perennially” moist (WFP > 80%; Figure 3) subsoils can promote CO_2_ dissolution and carbonic acid formation, providing a potential pathway through which deep CO_2_ accumulation contributes to mineral weathering (Keller 2019; Oh and Richter Jr. 2004) and aqueous inorganic‐carbon retention (Zamanian et al. 2024, 2016). Thus, soil moisture exerts competing controls on the fate of respired C by functioning as a promoter of root and microbial respiration at intermediate levels, but also potentially as a consumer at (near)saturation via gaseous CO_2_ removal (Billings et al. 2024; Sánchez‐Cañete et al. 2018; Oh and Richter Jr. 2004). These hydrochemical conversions represent active carbon transformation pathways, shifting CO_2_ from a volatile atmospheric constituent to a reactive agent via H_2_CO_3_ formation (Tetteh et al. 2025; Oh and Richter Jr. 2004) or a potential longer‐lived geochemical pool via bicarbonate formation and subsequent pedogenic carbonation (Tetteh et al. 2026; Kramer and Chadwick 2018). In this fundamental respect, respired CO_2_ emerges as the central currency of the soil carbon‐transformation continuum, produced by soil life and transformed through interaction with water into dissolved carbon species that may favor (de)carbonation under changing redox conditions (Guo et al. 2026; Hu et al. 2026), positioning respiration‐induced elevated subsoil CO_2_ pressures as regulators of porewater acidity (Tetteh et al. 2025; Hasenmueller et al. 2015; Oh and Richter Jr. 2004). Such processes provide a potential geochemical extension of the soil carbon cycle, informing carbon sequestration strategies and climate‐resilient land management. In deep‐rooting ecosystems, such as semi‐arid forests, and under hydrological settings where deeper groundwater tables do not constrain rooting and subsoil temperatures are favorable, deep carbonation processes may be more pronounced (Rowan et al. 2025; Zamanian 2024; Zamanian et al. 2016), particularly in well‐structured clay domains where (macro)pore connectivity can promote deeper CO_2_ loading (Oh and Richter Jr. 2004).

Consistent field observations and model simulations demonstrate that CO_2_ enrichment below the most bioactive topsoil arises predominantly from transport‐limited accumulation rather than in situ respiration alone (Figures 5 and 6), supporting emerging evidence for subsoil exchange of topsoil‐respired carbon (Wordell‐Dietrich et al. 2020; Oh et al. 2005; Oh and Richter Jr. 2004; Davidson and Trumbore 1995). Because soil CO_2_ concentration dynamics, the primary determinant of surface flux, emerge from profile‐scale biological CO_2_ production (Figure 5) and its transport and diffusivity processes operating across tightly coupled spatial and temporal scales under changing environmental constraints (Figures 3 and 4), high production does not necessarily translate into proportional CO_2_ exchange at the soil‐atmosphere boundary (Figure 6). Hence, interpretations based solely on surface measurements or isolated depth observations can be confounded by transport and storage processes (Lee et al. 2010; Holden and Fierer 2005; Reichstein and Beer 2008; Welles et al. 2001). This scale mismatch highlights a critical limitation in soil respiration research and necessitates model‐data integration frameworks that reconcile production, transport, and storage across scales (Figure 6) (Reichstein and Beer 2008; Šimůnek and Suarez 1993; Suarez and Šimůnek 1993). Within this context, our findings resolve the soil CO_2_ production‐transport imbalance, demonstrating that CO_2_ enrichment in wetter, colder, less bioactive subsoils (Figure 6) is governed by transport‐limited vertical redistribution (Figure 7) rather than in situ production alone (Figure 5).

These results provide a mechanistic basis for soil CO_2_ source strength and fate, showing that a substantial fraction of topsoil‐respired CO_2_ bypasses the soil‐atmosphere boundary and is redistributed downward, contributing to a substantial subsoil CO_2_‐C storage pool. Subsoils therefore function not merely as conduits but as active storage domains (Zamanian 2024), where accumulated CO_2_ (i.e., sum of influx and locally produced CO_2_) can reach concentrations up to two orders of magnitude higher than atmospheric levels, revealing a dominant subsurface pathway in soil respired carbon partitioning (Figure 6). This mechanism helps explain the long‐standing discrepancy between low subsoil respiration rates and disproportionately high CO_2_ concentrations and partial pressures at depth (Reichstein and Beer 2008; Lee et al. 2010). Consistent with emerging evidence (Sánchez‐Cañete et al. 2018; Lee et al. 2010; Oh et al. 2005), these findings further demonstrate that surface‐based soil CO_2_ assessment represents a transport‐limited, boundary‐conditioned signal (Figure 6) governed by evolving concentration gradients, pressure dynamics, and redistribution (Figure 7), rather than a direct proxy for total soil‐respired carbon. Consequently, reliance on surface measurements alone cannot resolve the full partitioning of soil‐respired carbon under field conditions. Incorporating depth‐dependent CO_2_ source strength and bidirectional exchange dynamics is therefore essential for accurately representing terrestrial carbon‐climate feedbacks, particularly under warming and land‐use change that intensify vertical coupling. Forest restoration, agroforestry, and structural soil conservation may thus enhance the transfer of photosynthetically fixed carbon into deeper soil through root‐derived inputs and respiration, followed by subsurface redistribution and retention of respired CO_2_, potentially reinforcing long‐term terrestrial carbon sinks (Billings et al. 2024; Zamanian et al. 2024; Keller 2019; Kramer and Chadwick 2018). Further investigations extending respired CO_2_ flux partitioning across atmospheric and groundwater boundaries and along hydrological gradients are required to quantify the magnitude and timing of the bidirectional exchange and persistence of CO_2_‐C retained in these deep domains and refine global estimates of the terrestrial carbon allocation.

## Author Contributions

**Kenneth Tetteh:** conceptualization, methodology, formal analysis, writing – original draft, data curation, investigation, visualization, software. **Mostafa Abdollahpour:** conceptualization, methodology, writing – review and editing, investigation, formal analysis, visualization. **Leopold Sauheitl:** conceptualization, methodology, writing – review and editing. **Georg Guggenberger:** conceptualization, methodology, writing – review and editing, data curation, project administration, supervision, resources, funding acquisition, validation. **Kazem Zamanian:** conceptualization, methodology, data curation, supervision, resources, project administration, funding acquisition, writing – review and editing, formal analysis.

## Funding

This work was supported by the Niedersächsisches Ministerium für Wissenschaft und Kultur (15‐76251‐2—Stay‐8/22‐5947/2022).

## Conflicts of Interest

The authors declare no conflicts of interest.

## Supporting information


**Figure S1:** Study area and site layout. (a) Location of the Fuhrberger Feld catchment (blue) northeast of Hannover, Germany (red, 30 km radius). (b) Experimental design showing three pairs of arable fields (A, gray), forest stands (F, green), and forest‐arable edges (E, orange). Numbered sites (1–3) represent topographic replicates yielding triplet locations comprising nine individual sampling sites (Agric 1–3, Forest 1–3, Edge 1–3).
**Figure S2:** Depth‐resolved (a) bulk density and (b) pH across land uses from 0–200 cm.
**Figure S3:** Depth profiles of soil organic carbon (SOC) stocks across cropland (a), forest‐cropland edges (b) and forests (c), illustrating increasing substrate scarcity with depth.
**Figure S4:** Root biomass density across croplands (a), forest‐cropland edges (b), and forests (c).
**Figure S5:** Depth profiles of soil microbial biomass carbon (MBC) stocks across croplands (a), forest‐cropland edges (b), and forests (c).
**Figure S6:** Daily‐to‐seasonal (Aug 2024‐Jul 2025) soil moisture (θ) dynamics across croplands (a), forest‐cropland edges (b) and forests (c).
**Table S1:** Soil texture by soil layer for cropland (Agric), edge, and forest sites at Fuhrberger Feld, Hannover, Germany. All soils are classified as sand according to USDA texture classes, with sand contents exceeding 85%–90% across depths and land‐use types.
**Table S2:** Soil hydraulic parameters (*θ*r, *θ*s, *α*, *n*, *K*_*s*_) and bimodal fitting parameters (w_2_, α_2_, n_2_) estimated for agricultural, edge, and forest soils across six depth increments (0–300 cm). Parameters were derived using LABROS SoilView Analysis software (version 5.3.0.0; HYPROP, METER Group GmbH, Germany) and applied in the DeepSoilBreath2 framework to represent depth‐specific retention and conductivity functions. Model performance was assessed with RMSE for water retention (RMSEθ), hydraulic conductivity (RMSEk), and the corrected Akaike Information Criterion (AICc).
**Table S3:** Organic matter (SOM) fraction of soil layers, used in the DeepCO_2_ framework to parameterize the volumetric share of organic matter controlling soil heat capacity and conductivity.
**Table S4:** Solid fraction (SF) of soil layers, used in the DeepCO_2_ framework to parameterize the mineral volume share governing soil thermal inertia and heat transfer.
**Table S5:** Exponential coefficients (*α*) describing the vertical distribution of soil CO_2_ production used in the DeepCO_2_ framework.

## Data Availability

The datasets supporting this study, including water‐filled porosity, soil temperature, soil CO_2_ production, and CO_2_ transport, effective CO_2_ diffusivity, are publicly available at Zenodo: https://doi.org/10.5281/zenodo.21920823.
