# Supplementary material for: Previously Unrecognized Land‐Use Control on Deep Soil CO2 Sink
Source: Glob Chang Biol. 2026 Sep 28;32(10):e71119. doi: 10.1111/gcb.71119 (PMC13618454; doi:10.1111/gcb.71119)
Supplement: Supplementary file 1 [file GCB-32-e71119-s001.docx]

**Supporting information for the manuscript:**

**Previously Unrecognized Land-Use Control on Deep Soil CO_2_ Sink**

Kenneth Tetteh^1*^, Mostafa Abdollahpour^1^, Leopold Sauheitl^1^, Georg Guggenberger^1^, Kazem Zamanian^1*^

1. Institute of Earth System Sciences, Section Soil Science, Leibniz University Hannover, Herrenhäuser Straße 2, 30419, Hannover, Germany

* Corresponding authors: [tetteh@ifbk.uni-hannover.de](mailto:tetteh@ifbk.uni-hannover.de), [zamanians@yahoo.com](mailto:zamanians@yahoo.com)

This file includes:

- Number of pages: 10
- Number of figures: 6
- Number of tables: 5

Contents: Page

- Figure S1: Study area and site layout	S1
- Figure S2: Depth-resolved bulk density and soil pH across land-use types	S2
- Figure S3: Depth profiles of soil organic carbon stocks across land-use	S2
- Figure S4: Depth profiles of root biomass density across land-use	S4
- Figure S5: Depth profiles of microbial biomass carbon stocks across land-use	S4-S5
- Figure S6: Soil moisture (θ) dynamics across land-use types	S5
- Tables S1-S5: Soil texture, hydraulic, thermal, and biogeochemical properties used in the DeepCO_2_ framework. S5-S10

b

a

2

3

1

**Figure S1.** Study area and site layout. (a) Location of the Fuhrberger Feld catchment (blue) northeast of Hannover, Germany (red, 30 km radius). (b) Experimental design showing three pairs of arable fields (A, gray), forest stands (F, green), and forest-arable edges (E, orange). Numbered sites (1-3) represent topographic replicates yielding triplet locations comprising nine individual sampling sites (Agric 1-3, Forest 1-3, Edge 1-3).

b

a

**Figure S2.** Depth-resolved (a) bulk density and (b) pH across land uses from 0-200 cm.

c

b

a

**Figure S3.** Depth profiles of soil organic carbon (SOC) stocks across cropland (a), forest-cropland edges (b) and forests (c), illustrating increasing substrate scarcity with depth.

c

b

a

**Figure S4.** Root biomass density across croplands (a), forest-cropland edges (b), and forests (c).

**Figure S5.** Depth profiles of soil microbial biomass carbon (MBC) stocks across croplands (a), forest-cropland edges (b), and forests (c).

a

c

b

c

b

a

**Figure S6.** Daily-to-seasonal (Aug 2024-Jul 2025) soil moisture (θ) dynamics across croplands (a), forest-cropland edges (b) and forests (c).

**Table S1.** Soil texture by soil layer for cropland (Agric), edge, and forest sites at Fuhrberger Feld, Hannover, Germany. All soils are classified as sand according to USDA texture classes, with sand contents exceeding 85-90% across depths and land-use types.

| Site / Land use | Coordinates (WGS84) | Soil layer (cm) | Clay (%) | Silt (%) | Sand (%) |
| --- | --- | --- | --- | --- | --- |
| Agric 1 | 52.56791° N, 9.86610° E | 0-30 | 1.10 | 5.60 | 93.30 |
|  |  | 30-50 | 4.20 | 9.10 | 86.70 |
|  |  | 50-100 | 3.00 | 4.20 | 92.70 |
|  |  | 100-200 | 1.90 | 0.10 | 98.00 |
| Edge 1 | 52.56791° N, 9.86610° E | 0-30 | 1.60 | 4.80 | 93.60 |
|  |  | 30-50 | 0.70 | 1.50 | 97.80 |
|  |  | 50-100 | 0.80 | 1.90 | 97.40 |
|  |  | 100-200 | 0.80 | 2.20 | 97.00 |
| Forest 1 | 52.57158° N, 9.85448° E | 0-30 | 0.00 | 4.00 | 96.00 |
|  |  | 30-50 | 0.00 | 4.80 | 95.20 |
|  |  | 50-100 | 0.00 | 3.90 | 96.00 |
|  |  | 100-200 | 0.00 | 5.80 | 94.20 |
| Agric 2 | 52.54724° N, 9.84007° E | 0-30 | 1.05 | 4.85 | 94.10 |
|  |  | 30-50 | 3.42 | 7.54 | 89.00 |
|  |  | 50-100 | 3.03 | 4.23 | 92.70 |
|  |  | 100-150 | 0.10 | 4.22 | 95.70 |
|  |  | 150-200 | 0.06 | 4.97 | 95.00 |
| Edge 2 | 52.54724° N, 9.84007° E | 0-30 | 3.33 | 3.97 | 92.70 |
|  |  | 30-50 | 1.12 | 3.65 | 95.20 |
|  |  | 50-100 | 0.91 | 0.70 | 98.40 |
|  |  | 100-200 | 1.05 | 0.39 | 98.60 |
| Forest 2 | 52.56014° N, 9.80626° E | 0-30 | 2.29 | 8.25 | 89.50 |
|  |  | 30-50 | 1.34 | 8.76 | 89.90 |
|  |  | 50-100 | 0.02 | 5.68 | 94.30 |
|  |  | 100-150 | 0.00 | 4.85 | 95.10 |
|  |  | 150-200 | 0.00 | 4.05 | 95.90 |
| Agric 3 | 52.58230° N, 9.82108° E | 0-30 | 2.11 | 5.98 | 91.90 |
|  |  | 30-50 | 2.17 | 7.97 | 89.90 |
|  |  | 50-100 | 0.84 | 3.25 | 95.90 |
|  |  | 100-150 | 0.60 | 3.40 | 96.00 |
|  |  | 150-200 | 0.34 | 2.55 | 97.10 |
| Edge 3 | 52.58230° N, 9.82108° E | 0-30 | 0.53 | 9.96 | 89.50 |
|  |  | 30-50 | 0.32 | 7.71 | 92.00 |
|  |  | 50-100 | 0.50 | 3.40 | 96.10 |
|  |  | 100-150 | 0.27 | 3.39 | 96.30 |
|  |  | 150–200 | 0.23 | 3.39 | 96.40 |
| Forest 3 | 52.58624° N, 9.81722° E | 0-30 | 1.42 | 3.72 | 94.90 |
|  |  | 30-50 | 0.47 | 4.52 | 95.00 |
|  |  | 50-100 | 0.11 | 3.94 | 96.00 |
|  |  | 100-150 | 0.07 | 3.52 | 96.40 |
|  |  | 150-200 | 0.06 | 3.51 | 96.40 |

**Table S2.** Soil hydraulic parameters (θr, θs, α, n, Ks) and bimodal fitting parameters (w₂, α₂, n₂) estimated for agricultural, edge, and forest soils across six depth increments (0-300 cm). Parameters were derived using LABROS SoilView Analysis™ software (version 5.3.0.0; HYPROP©, METER Group GmbH, Germany) and applied in the DeepSoilBreath2 framework to represent depth-specific retention and conductivity functions. Model performance was assessed with RMSE for water retention (RMSEθ), hydraulic conductivity (RMSEk), and the corrected Akaike Information Criterion (AICc).

| Land use | Layer θr θs α_1_ n_1_ ks w_2_ α_2_ n_2_ RMSEθ RMSEks AICc  (cm) (cm^3^ cm^-3^) (cm^3^ cm^-3^) (cm^-1^) (-) (cm d^-1^) (-) (cm^-1^) (-) (cm^3^ cm^-3^) (cm d^-1^) | | | | | | | | | | | |
| --- | --- | --- | --- | --- | --- | --- | --- | --- | --- | --- | --- | --- |
| Agric 1 | 0-10 | 0.039 | 0.442 | 0.024 | 1.74 | 218 | 0.447 | 0.031 | 6.42 | 0.022 | 0.114 | -1051 |
|  | 10-30 | 0.034 | 0.413 | 0.021 | 1.64 | 178 | 0.407 | 0.028 | 6.21 | 0.021 | 0.122 | -1046 |
|  | 30-50 | 0.025 | 0.347 | 0.007 | 1.25 | 65.1 | 0.625 | 0.016 | 5.04 | 0.005 | 0.273 | -1301 |
|  | 50-100 | 0.028 | 0.377 | 0.014 | 1.37 | 81.5 | 0.327 | 0.024 | 5.78 | 0.013 | 0.122 | -1051 |
|  | 100-150 | 0.027 | 0.366 | 0.017 | 1.34 | 74.4 | 0.325 | 0.022 | 5.83 | 0.017 | 0.126 | -1056 |
|  | 150-200 | 0.029 | 0.362 | 0.015 | 1.39 | 72.1 | 0.319 | 0.028 | 5.87 | 0.011 | 0.124 | -1062 |
| Agric 2 | 0-10 | 0.039 | 0.453 | 0.029 | 1.62 | 713 | 0.406 | 0.034 | 4.75 | 0.006 | 0.155 | -1319 |
|  | 10-30 | 0.034 | 0.423 | 0.025 | 1.52 | 249 | 0.366 | 0.03 | 4.55 | 0.006 | 0.163 | -1314 |
|  | 30-50 | 0.045 | 0.415 | 0.029 | 3.53 | 89.2 | 0.496 | 0.015 | 1.25 | 0.006 | 0.186 | -1217 |
|  | 50-100 | 0.028 | 0.381 | 0.016 | 1.26 | 204 | 0.287 | 0.022 | 4.15 | 0.005 | 0.172 | -1324 |
|  | 100-150 | 0.032 | 0.37 | 0.019 | 1.28 | 81.4 | 0.284 | 0.021 | 4.15 | 0.005 | 0.172 | -1324 |
|  | 150-200 | 0.029 | 0.362 | 0.017 | 1.27 | 76.6 | 0.286 | 0.024 | 4.15 | 0.005 | 0.172 | -1324 |
| Agric 3 | 0-10 | 0.035 | 0.509 | 0.023 | 1.9 | 252 | 0.49 | 0.022 | 4.2 | 0.011 | 0.19 | -1205 |
|  | 10-30 | 0.03 | 0.472 | 0.02 | 1.8 | 200 | 0.45 | 0.02 | 4 | 0.01 | 0.2 | -1200 |
|  | 30-50 | 0.026 | 0.396 | 0.017 | 1.6 | 88.9 | 0.4 | 0.017 | 3.7 | 0.009 | 0.22 | -1215 |
|  | 50-100 | 0.024 | 0.389 | 0.013 | 1.55 | 67.1 | 0.37 | 0.015 | 3.6 | 0.008 | 0.21 | -1210 |
|  | 100-150 | 0.027 | 0.396 | 0.015 | 1.57 | 71.1 | 0.35 | 0.014 | 3.6 | 0.008 | 0.21 | -1210 |
|  | 150-200 | 0.025 | 0.389 | 0.017 | 1.58 | 67.1 | 0.36 | 0.016 | 3.6 | 0.008 | 0.21 | -1210 |
| Edge 1 | 0-10 | 0.04 | 0.485 | 0.021 | 1.77 | 127 | 0.484 | 0.029 | 5.54 | 0.007 | 0.245 | -1391 |
|  | 10-30 | 0.035 | 0.418 | 0.018 | 1.67 | 204 | 0.444 | 0.026 | 5.34 | 0.006 | 0.258 | -1386 |
|  | 30-50 | 0.031 | 0.377 | 0.014 | 1.47 | 52.5 | 0.394 | 0.02 | 5.04 | 0.006 | 0.283 | -1401 |
|  | 50-100 | 0.03 | 0.374 | 0.012 | 1.42 | 55.5 | 0.366 | 0.018 | 4.92 | 0.005 | 0.27 | -1396 |
|  | 100-150 | 0.029 | 0.366 | 0.012 | 1.41 | 52.2 | 0.364 | 0.017 | 4.94 | 0.005 | 0.27 | -1396 |
|  | 150-200 | 0.029 | 0.358 | 0.011 | 1.43 | 49.1 | 0.365 | 0.019 | 4.95 | 0.005 | 0.27 | -1396 |
| Edge 2 | 0-10 | 0.035 | 0.472 | 0.025 | 3.94 | 151 | 0.54 | 0.004 | 1.62 | 0.011 | 0.105 | -1151 |
|  | 10-30 | 0.03 | 0.442 | 0.022 | 3.84 | 124 | 0.5 | 0.003 | 1.42 | 0.01 | 0.11 | -1146 |
|  | 30-50 | 0.026 | 0.396 | 0.015 | 3.64 | 67.2 | 0.45 | 0.003 | 1.12 | 0.009 | 0.121 | -1161 |
|  | 50-100 | 0.024 | 0.381 | 0.014 | 3.59 | 47.9 | 0.41 | 0.003 | 1.13 | 0.008 | 0.116 | -1156 |
|  | 100-150 | 0.025 | 0.374 | 0.014 | 3.61 | 45.1 | 0.44 | 0.003 | 1.15 | 0.008 | 0.116 | -1156 |
|  | 150-200 | 0.027 | 0.366 | 0.017 | 3.58 | 42.4 | 0.42 | 0.003 | 1.14 | 0.008 | 0.116 | -1156 |
| Edge 3 | 0-10 | 0.04 | 0.434 | 0.015 | 2.94 | 369 | 0.607 | 0.038 | 13.3 | 0.004 | 0.26 | -1422 |
|  | 10-30 | 0.035 | 0.426 | 0.013 | 2.84 | 350 | 0.567 | 0.034 | 13.1 | 0.004 | 0.274 | -1417 |
|  | 30-50 | 0.031 | 0.389 | 0.009 | 2.64 | 146 | 0.517 | 0.026 | 12.8 | 0.003 | 0.301 | -1432 |
|  | 50-100 | 0.029 | 0.374 | 0.009 | 2.59 | 141 | 0.487 | 0.024 | 12.7 | 0.003 | 0.287 | -1427 |
|  | 100-150 | 0.03 | 0.366 | 0.009 | 2.59 | 133 | 0.487 | 0.024 | 12.7 | 0.003 | 0.287 | -1427 |
|  | 150-200 | 0.032 | 0.358 | 0.009 | 2.59 | 125 | 0.487 | 0.024 | 12.7 | 0.003 | 0.287 | -1427 |
| Forest 1 | 0-10 | 0.024 | 0.453 | 0.025 | 1.7 | 342 | 0.674 | 0.038 | 4.3 | 0.013 | 0.489 | -956 |
|  | 10-30 | 0.019 | 0.457 | 0.022 | 1.6 | 350 | 0.634 | 0.034 | 4.1 | 0.012 | 0.515 | -951 |
|  | 30-50 | 0.015 | 0.389 | 0.015 | 1.4 | 119 | 0.584 | 0.026 | 3.8 | 0.01 | 0.567 | -966 |
|  | 50-100 | 0.015 | 0.374 | 0.014 | 1.35 | 115 | 0.554 | 0.024 | 3.7 | 0.01 | 0.541 | -961 |
|  | 100-150 | 0.015 | 0.366 | 0.014 | 1.35 | 108 | 0.554 | 0.024 | 3.7 | 0.01 | 0.541 | -961 |
|  | 150-200 | 0.015 | 0.358 | 0.014 | 1.35 | 102 | 0.554 | 0.024 | 3.7 | 0.01 | 0.541 | -961 |
| Forest 2 | 0-10 | 0.041 | 0.491 | 0.043 | 6.09 | 463 | 0.419 | 0.009 | 2.36 | 0.032 | 0.105 | -960 |
|  | 10-30 | 0.036 | 0.447 | 0.038 | 5.99 | 350 | 0.379 | 0.008 | 2.16 | 0.029 | 0.111 | -955 |
|  | 30-50 | 0.032 | 0.404 | 0.026 | 5.79 | 194 | 0.329 | 0.006 | 1.86 | 0.026 | 0.122 | -970 |
|  | 50-100 | 0.03 | 0.389 | 0.025 | 5.74 | 138 | 0.299 | 0.006 | 1.76 | 0.024 | 0.116 | -965 |
|  | 100-150 | 0.03 | 0.377 | 0.025 | 5.74 | 126 | 0.299 | 0.006 | 1.76 | 0.024 | 0.116 | -965 |
|  | 150-200 | 0.03 | 0.37 | 0.025 | 5.74 | 119 | 0.299 | 0.006 | 1.76 | 0.024 | 0.116 | -965 |
| Forest 3 | 0-10 | 0.057 | 0.698 | 0.009 | 2.28 | 828 | 0.562 | 0.044 | 3.22 | 0.011 | 0.188 | -1309 |
|  | 10-30 | 0.052 | 0.524 | 0.008 | 2.18 | 350 | 0.522 | 0.04 | 3.02 | 0.01 | 0.198 | -1304 |
|  | 30-50 | 0.048 | 0.385 | 0.006 | 1.98 | 76.3 | 0.472 | 0.03 | 2.72 | 0.009 | 0.217 | -1319 |
|  | 50-100 | 0.046 | 0.389 | 0.005 | 1.93 | 85.8 | 0.442 | 0.028 | 2.62 | 0.008 | 0.208 | -1314 |
|  | 100-150 | 0.046 | 0.381 | 0.005 | 1.93 | 80.9 | 0.442 | 0.028 | 2.62 | 0.008 | 0.208 | -1314 |
|  | 150-200 | 0.046 | 0.374 | 0.005 | 1.93 | 76.1 | 0.442 | 0.028 | 2.62 | 0.008 | 0.208 | -1314 |

**Table S3.** Organic matter (SOM) fraction of soil layers, used in the DeepCO_2_ framework to parameterize the volumetric share of organic matter controlling soil heat capacity and conductivity.

| Layer (cm) | Agric 1 | Agric 2 | Agric 3 | Edge 1 | Edge 2 | Edge 3 | Forest 1 | Forest 2 | Forest 3 |
| --- | --- | --- | --- | --- | --- | --- | --- | --- | --- |
| 0-10 | 0.0292 | 0.0388 | 0.0543 | 0.0332 | 0.0457 | 0.0313 | 0.0113 | 0.0429 | 0.0364 |
| 10-30 | 0.0297 | 0.0483 | 0.0601 | 0.0378 | 0.0468 | 0.0313 | 0.0112 | 0.0427 | 0.0437 |
| 30-50 | 0.003 | 0.0108 | 0.0318 | 0.0098 | 0.0298 | 0.007 | 0.0024 | 0.008 | 0.0041 |
| 50-100 | 0.003 | 0.002 | 0.0032 | 0.0083 | 0.0123 | 0.0037 | 0.0025 | 0.0085 | 0.002 |
| 100-150 | 0.0024 | 0.002 | 0.0157 | 0.0115 | 0.0077 | 0.0072 | 0.0025 | 0.0025 | 0.0036 |
| 150-200 | 0.0023 | 0.0013 | 0.002 | 0.0116 | 0.0077 | 0.0041 | 0.0025 | 0.0044 | 0.0053 |

**Table S4.** Solid fraction (SF) of soil layers, used in the DeepCO_2_ framework to parameterize the mineral volume share governing soil thermal inertia and heat transfer.

| Layer(cm) | Agric 1 | Agric 2 | Agric 3 | Edge 1 | Edge 2 | Edge 3 | Forest 1 | Forest 2 | Forest 3 |
| --- | --- | --- | --- | --- | --- | --- | --- | --- | --- |
| 0-10 | 0.5268 | 0.4622 | 0.4981 | 0.4243 | 0.4742 | 0.5736 | 0.5455 | 0.5088 | 0.3019 |
| 10-30 | 0.5357 | 0.5765 | 0.5519 | 0.483 | 0.4864 | 0.5736 | 0.5434 | 0.5067 | 0.3625 |
| 30-50 | 0.6513 | 0.5846 | 0.5847 | 0.6209 | 0.5651 | 0.5887 | 0.5914 | 0.557 | 0.6138 |
| 50-100 | 0.58 | 0.6119 | 0.5743 | 0.5995 | 0.5814 | 0.5321 | 0.5793 | 0.5886 | 0.5553 |
| 100-150 | 0.6449 | 0.637 | 0.5726 | 0.6102 | 0.6056 | 0.5547 | 0.5889 | 0.5794 | 0.5782 |
| 150-200 | 0.6056 | 0.6193 | 0.5064 | 0.6155 | 0.6151 | 0.6038 | 0.6129 | 0.628 | 0.5971 |

**Table S5.** Exponential coefficients (*α*) describing the vertical distribution of soil CO_2_ production used in the DeepCO_2_ framework.

| Land Use | Agric 1 | Agric 2 | Agric 3 | Edge 1 | Edge 2 | Edge 3 | Forest 1 | Forest 2 | Forest 3 |
| --- | --- | --- | --- | --- | --- | --- | --- | --- | --- |
| *a* (cm^-1^) | 0.0511 | 0.0513 | 0.0553 | 0.0725 | 0.0734 | 0.0769 | 0.0043 | 0.0441 | 0.0453 |
